# The Potential Interplay of Adipokines with Toll-Like Receptors in the Development of Hepatocellular Carcinoma

**DOI:** 10.1155/2011/215986

**Published:** 2011-09-25

**Authors:** Shen-Nien Wang, Sen-Te Wang, King Teh Lee

**Affiliations:** ^1^Department of Surgery, Faculty of Medicine, College of Medicine, Kaohsiung Medical University, Kaohsiung, Taiwan; ^2^Graduate Institute of Medicine, College of Medicine, Kaohsiung Medical University, Kaohsiung, Taiwan; ^3^Cancer Center, Kaohsiung Medical University Hospital, Kaohsiung, Taiwan; ^4^Department of Family Medicine, School of Medicine, College of Medicine, Taipei Medical University, Taipei, Taiwan; ^5^Department of Family Medicine, Taipei Medical University Hospital, Taipei, Taiwan; ^6^Division of Hepatobiliary Surgery, Department of Surgery, Kaohsiung Medical University Hospital, No. 100, Shih-Chuan 1st Road, San Ming District, Kaohsiung 80756, Taiwan

## Abstract

Toll-like receptors (TLRs) are not only crucial to the initiation of the immune system, but also play a key role in several human inflammatory diseases. Hepatocellular carcinoma (HCC) is among those human cancers, which arise from sites of chronic inflammation. Therefore, a number of studies have explored the potential contribution of TLRs to HCC occurrence, which is initiated by exposure to chronic hepatic inflammation of different etiologies (including ethanol, and chronic B and C viral infections). Recent epidemiological data have shown the association of obesity and HCC development. Given the fact that adipose tissues can produce a variety of inflammation-related adipokines, obesity has been characterized as a state of chronic inflammation. Adipokines are therefore considered as important mediators linking inflammation to several metabolic diseases, including cancers. More recently, many experts have also shown the bridging role of TLRs between inflammation and metabolism. Hopefully, to retrieve the potential interaction between TLRs and adipokines in carcinogenesis of HCC will shed a new light on the therapeutic alternative for HCC. In this paper, the authors first review the respective roles of TLRs and adipokines, discuss their mutual interaction in chronic inflammation, and finally anticipate further investigations of this interaction in HCC development.

## 1. Overviews of Toll-Like Receptors (TLRs)

Since *Drosophila *Toll was found to resemble the amino acid sequence of the cytoplasmic portion of interleukin-1 (IL-1), accumulated studies have unveiled the crucial role of Toll-like receptors (TLRs) in induction of innate immune responses [1, 2]. Host cells express a variety of pattern recognition receptors (PRRs) that recognize diverse pathogen-associated molecular patterns (PAMPs), including lipids, lipoproteins, proteins, and nucleic acids [3, 4]. TLRs belong to the family of type I transmembrane receptors characterized by an extracellular leucine rich repeat (LRR) domain that is responsible for recognition of PAMPs and an intracellular domain homologous to the cytoplasmic region of the IL-1 receptor (known as TIR domain) that is required for downstream signaling [5, 6]. To date, at least eleven members of human TLR family have been identified and recognized different PAMPs derived from distinct microorganisms, including bacteria, viruses, protozoa, and fungi [7]. 

The TIR domain is required for the interaction and recruitment of various adaptor molecules to activate diverse downstream signaling pathways. Several TIR domain-containing adaptor molecules have been defined, including MyD88, MyD88-adaptor-like (MAL, also known as TIRAP), TIR-domain-containing adaptor protein inducing IFN*β* (TRIF; also known as TICAM1), TRIF-related adaptor molecule (TRAM; also known as TICAM2), and sterile *α*- and armadillo-motif-containing protein (SARM) [8–12]. Upon recognition of the cognate ligand, each TLR recruits a specific adaptor molecule to activate different transcription factors, giving rise to appropriate and effective responses to pathogens. 

MyD88 is a universal adaptor molecule that activates inflammatory pathways, and it is shared by all TLRs with the exception of TLR3. The association of TLRs and MyD88 stimulates the recruitment of members of the IRAK family, including IRAK1, IRAK2, IRAK4, and IRAK-M. In particular, IRAK4 is indispensable for activation of the MyD88-dependent pathway [13]. Once phosphorylated, IRAKs dissociate from MyD88 and interact with TRAF6, which serves as a platform to recruit the kinase TAK1 [14]. Once activated, TAK1 activates MAP kinases (ERK, JNK, p38) and the transcription factor NF-**κ**B to control the expression of inflammatory cytokine genes. TIRAP mediates the activation of a MyD88-dependent pathway downstream of TLR2 and TLR4. On the other hand, TLR3 activates MyD88-independent signaling, indicating the importance of other TIR domain-containing adaptor molecules that function in the TLR3- and TLR4-mediated pathways [15]. TRIF plays a critical role in the TLR3- and TLR4-mediated MyD88-independent pathways [10]. TRIF directly engages with TLR3, whereas TRAM bridges the TLR4 interaction [16]. TRIF cascade results in activation of IRF3. Phosphorylated IRF3 dimerizes and is translocated to the nucleus to induce expression of type I IFN and IFN-inducible genes. Briefly, activation of TLR signaling culminates in NF-**κ**B and MAPKs that regulate gene expression of various immune and inflammatory mediators.

## 2. The Involvement of TLRs in Inflammation-Triggered HCC

TLRs are not only crucial to the initiation of the innate and adaptive immune reactions, but also play a key role in chronic inflammation. Aberrant activation of TLR pathways has been implicated in a variety of chronic and autoimmune diseases [17–19]. Also, inflammation is an important trigger for carcinogenesis of several human cancers. The causal relationship of chronic inflammation with cancer development was first mentioned by Rudolph Virchow in 1863, who observed that tumors frequently developed on sites of sustained inflammation and tumoral tissues were often infiltrated by a variety of inflammatory cells [20]. Hepatocellular carcinoma (HCC) is a typical example of inflammation-related cancer, which slowly unfolds on a background of chronic inflammation mainly triggered by exposure to infectious agents (chronic hepatitis B and C viral infection) and toxic compounds (ethanol). 

Many different cell types in the liver express TLRs [21]. Hepatocytes uptake and eliminate endotoxin from portal and systemic circulation. Primary cultured hepatocytes express mRNA for all TLRs and respond to TLR2 and TLR4 ligands [22]. Kupffer cells, hepatic resident macrophages, are located in hepatic sinusoids and are the principal liver cells for phagocytosis, antigen presentation, and the production of inflammatory cytokines. Because of the unique anatomical link between the liver and intestines, Kupffer cells are the first cells to encounter gut-derived toxins including LPS, but are less responsive by “LPS tolerance” under the physiological environment. Upon triggering, TLR4 signaling drives Kupffer cells to produce a variety of inflammation-related cytokines [23]. Kupffer cells also express TLR2, TLR3, and TLR9 [24]. Hepatic stellate cells (HSCs) are located in the space of Disse. Activated HSCs transdifferentiate into myofiboblasts, which then produce excessive ECM proteins, including collagen type I, III, and IV. This leads to an irreversible collagen deposition, resulting in liver fibrosis. HSCs express TLR4 and TLR9 [25, 26]. Biliary epithelial cells may contact enteric bacteria by the anatomical linking between the biliary system and the intestinal lumen. Biliary epithelial cells express TLR2, TLR3, TLR4, and TLR5 [27]. Hepatic sinusoids are lined by liver sinusoidal endothelial cells that express TLR4 [28].

Aberrant TLRs expressions have been noted in chronic liver diseases of different etiology. In chronic hepatitis B virus (HBV) infection, the injection of ligands for TLR3, TLR4, TLR5, TLR7, and TLR9 suppresses HBV replication in an IFN-*α*/*β*-dependent manner in HBV transgenic mice [29]. Further experiments demonstrated that nonparenchymal liver cell-derived mediators inhibit HBV replication in HBV-Met cells [30]. These data indicate that the innate immune system of the liver can control HBV replication after activation by TLR agonists. Hepatitis C virus (HCV) evades the host immune system to sustain a chronic infection. Chronic HCV infection causes chronic liver inflammation and fibrosis, resulting in cirrhosis and HCC. TLR2 and TLR4 are markedly upregulated in hepatocytes, Kupffer cells, and peripheral monocytes of patients with chronic hepatitis C. TLR2-mediated activation by hepatitis C is linked to the proinflammatory cytokine induction [31]. The HCV core and NS3 protein activate TLR2/TLR1 and TLR2/TLR6 on monocytes to produce inflammatory cytokines [21]. Excessive alcohol intake changes the intestinal epithelial barrier causing increased intestinal permeability followed by elevated LPS levels in the portal circulation. The LPS then activates TLR4 on Kupffer cells to produce proinflammatory cytokines, such as TNF-*α*, leading to hepatocyte damage [32]. In addition, Kupffer cells produce reactive oxygen species (ROS) in response to chronic alcohol exposure as well as endotoxin. Evidence shows that direct interaction of NADPH oxidase isozyme 4 with TLR4 is involved in LPS-mediated ROS generation and NF*κ*B activation in neutrophils [33].

## 3. Adipokine: A Key Role in Obesity-Related Inflammation

Since obesity has become an epidemic health issue, researchers have found that obesity, particularly abdominal obesity, is associated with resistance to the effects of insulin on peripheral glucose and fatty acid utilization, often leading to type 2 diabetes mellitus. Moreover, long-term status of insulin resistance may cause vascular endothelial dysfunction, an abnormal lipid profile, hypertension, and vascular inflammation, all of which predispose to the development of atherosclerotic cardiovascular disease (CVD). Therefore, “metabolic syndrome” is defined to describe the strong association of obesity with these metabolic disorders. Indeed, accumulating evidence has described a clear connection between low-grade inflammatory responses and the development of metabolic diseases, particularly in the context of obesity and type 2 diabetes [34, 35]. Further investigations show that obese persons have increased levels of C-reactive protein (CRP), one proinflammatory marker in the blood [36]. In addition, increased levels of CRP, and its inducer interleukin-6 (IL-6), are predictive of the development of type 2 diabetes in various populations [37].

Adipose tissue was traditionally considered to be a long-term energy storage organ, but it is now appreciated that it has a key role in the integration of systemic metabolism [38]. This metabolic function is mediated, in a large part, by its ability to secrete numerous proteins. Factors that are secreted by adipose tissue have been collectively referred to as adipokines. Adipokines have both proinflammatory and anti-inflammatory activities, and the balance between these factors is crucial for determining homeostasis throughout the body based on nutritional status. When adipocyte dysfunction occurs as a result of adipose tissue expansion (which may be due to overnutrition or physical inactivity, for example), dysregulation of adipokine production can have local or systemic effects on inflammatory responses, thereby contributing to the initiation and progression of obesity-induced metabolic and cardiovascular complications [39]. 

### 3.1. Leptin

Leptin is the product of the *obese* (*ob*) gene, was first identified in *ob/ob *mice in 1994 [40]. Leptin is an adipocyte-derived hormone/cytokine that links nutritional status with neuroendocrine and immune functions. As a hormone, leptin regulates feeding behaviour through the central nervous system. Mice that lack leptin (*ob/ob *mice) show hyperphagia (abnormally increased feeding), obesity and insulin resistance, and the administration of leptin to *ob/ob *mice reverses these changes [41]. As a cytokine, leptin can affect thymic homeostasis and the secretion of acute-phase reactants such as IL-1 and TNF, suggesting its modulating role between immunity and inflammation. Leptin belongs to the family of long-chain helical cytokines (characterized by a four *α*-helix bundle) [42]. Leptin has structural similarity to IL-6, IL-12, IL-15, granulocyte colony-stimulating factor (G-CSF), and prolactin and growth hormone and is thought to have proinflammatory activities. Indeed, leptin increases the production of TNF and IL-6 by monocytes and stimulates the production of CC-chemokine ligands (namely, CCL3, CCL4, and CCL5) by macrophages by activating the JAK2 (Janus kinase 2)—STAT3 (signal transducer and activator of transcription 3) pathway [43, 44]. In monocytes, leptin also stimulates the production of ROs and promotes cell proliferation and migratory responses [45]. Leptin levels in the serum and adipose tissues are increased in response to proinflammatory stimuli, including TNF and lipopolysaccharide (LPS) [46].

### 3.2. Adiponectin

Adiponectin is mainly synthesized by adipocytes and is present at high levels (3 to 30 *μ*g/mL) in the blood [47]. Adiponectin is characterized by an amino-terminal collagen-like region and a carboxy-terminal, complement factor C1q-like globular domain. Serum levels of adiponectin are markedly decreased in individuals with visceral obesity and states of insulin resistance, such as nonalcoholic fatty liver disease, atherosclerosis and type 2 diabetes mellitus, and adiponectin levels correlate inversely with insulin resistance [48]. Much evidence from experimental models indicates that adiponectin protects against obesity-linked metabolic dysfunction. Administration of adiponectin to diabetic mice has been shown to reduce hyperglycaemia by enhancing insulin activity and, when given to obese mice, it increases fatty acid oxidation in muscle tissue and reduces plasma levels of glucose, free fatty acids, and triglycerides [49]. Early studies indicated that adiponectin had an anti-inflammatory effect on endothelial cells through the inhibition of TNF-induced adhesion-molecule expression [50]. Consistent with this, the production of adiponectin by adipocytes is inhibited by proinflammatory factors, such as TNF and IL-6, as well as by hypoxia and oxidative stress [51]. Also, adiponectin has a crucial role in suppressing macrophage activity, not only in adipose tissue but also in other tissues such as the liver. Adiponectin can induce the production of important anti-inflammatory cytokines, such as IL-10 and IL-1 receptor antagonist (IL-1RA), by human monocytes, macrophages, and dendritic cells and can also suppress the production of interferon-*γ* by LPS-stimulated human macrophages [52]. Recent evidence shows that globular adiponectin suppresses TLR-induced NF-*κ*B activation, indicating that adiponectin negatively regulates macrophage responses to TLR ligands, which is probably of relevance in innate immune responses [53].

### 3.3. Resistin

(Also Known as FIZZ3) Resistin is a 114-amino-acid polypeptide, was originally shown to induce insulin resistance in mice [54]. It is a member of the cysteine-rich family of resistin-like molecules (RELMs) that are associated with the activation of inflammatory processes [55]. In mice, resistin protein synthesis is restricted to adipocytes, whereas in humans, resistin is mainly produced by macrophages and monocytes, and it is not detectable in adipocytes [54, 56]. The proinflammatory properties of resistin in human mononuclear cells are evident, as resistin promotes the expression of TNF and IL-6 by these cells [57]. In addition, resistin directly counters the anti-inflammatory effects of adiponectin on vascular endothelial cells by promoting the expression of the proinflammatory adhesion molecules vascular cell adhesion molecule 1 (vCAm1), intercellular adhesion molecule 1 (ICAm1), and pentraxin 3 in these cells, thereby enhancing leukocyte adhesion [58, 59].

### 3.4. Visfatin

(Also Known as PBEF) Visfatin has recently been identified as an adipokine that is secreted by adipocytes in visceral fat and that decreases insulin resistance [60]. This molecule binds to and activates the insulin receptor but does not compete with insulin, which indicates that the two proteins bind different sites on the insulin receptor. Visfatin has been linked to several inflammatory disease states such as acute lung injury [61]. Moreover, expression of visfatin has been shown to be upregulated in activated neutrophils and to inhibit the apoptosis of neutrophils [62].

### 3.5. TNF

TNF is a proinflammatory cytokine that is mainly produced by monocytes and macrophages. TNF expression was found to be increased in the adipose tissues of experimental animal models of obesity and type 2 diabetes [63]. Further work demonstrated that TNF attenuated insulin-stimulated tyrosine phosphorylation of the insulin receptor and IRs1 in muscle and adipose tissues, thus promoting insulin resistance [64]. These data support the notion that TNF functions as a proinflammatory cytokine that has a crucial role in obesity-related insulin resistance. In fact, clinical data also showed that TNF levels were increased in the adipose tissue and plasma of obese individuals, and a reduction of body weight in these individuals was associated with a decrease in TNF expression [65, 66].

### 3.6. IL-6

IL-6, a proinflammatory cytokine, is also involved in obesity-related insulin resistance. It is estimated that approximately one-third of total circulating IL-6 is produced by adipose tissues, and it is possible that increased secretion of IL-6 under conditions of obesity contributes to metabolic dysfunction [67]. Clinical data indicated the positive correlation of plasma IL-6 levels with adiposity in human populations. Furthermore, plasma levels IL-6 levels were increased in patients with type 2 diabetes, and increased IL-6 levels were predictive of the development of type 2 diabetes [37].

## 4. The Interaction of Adipokines and Toll-Like Receptors: A Link between Inflammation and Metabolism

In adipose tissue, adipocytes are the main cellular components, and play a crucial role in both energy storage and endocrine activity. Other active cell components include macrophages and T cells, which have major roles in determining the immune status of adipose tissue. As obesity develops, adipocytes undergo hypertrophy owing to increased triglyceride storage. Once qualitative changes occur in the expanding adipose tissue, adipocytes will be transformed to a metabolically dysfunctional phenotype. In this state, adipose tissue generates large amounts of proinflammatory adipokines, which will lead to the recruitment and modulation of macrophages and other immune cells. Therefore, obesity has recently been characterized by low grade and chronic inflammation and is further defined as metaflammation [68]. 

Evidence has advocated that adipocytes may play a modulator role between inflammation and metabolic disorders via TLR signaling cascades. Lin et al. were successful in cloning Toll-like receptor-2 (TLR2) from 3T3-L1 adipocytes, and they showed that adipocytic TLR2 synthesis increases upon stimulation with LPS and tumor necrosis factor (TNF), respectively [69]. Mesenchymal stem cells that were isolated from human adipose tissue were reported to express TLR1 to TLR6 and TLR9 [70]. Stimulation with bacterial LPS, yeast cell wall zymosan, or TNF leads to a dramatic induction of TLR2 synthesis [71]. Collectively, it is assumable that adipokines might modulate obesity-related inflammatory responses through TLR signaling.

Given the fact that leptin plays a regulatory role in the immune system [72], recent study demonstrated the expression and responsiveness of TLR1 to -9 in murine preadipocytes as well as adipocytes, both of which are strongly regulated by leptin [73]. In addition, ob/ob mice (leptin-deficient obese mice) and db/db mice (mice lacking the long and functional isoform of the leptin receptor) show the broadest mRNA expression profile of TLRs in preadipocytes and mature adipocytes. Recent data showed that the obesity induced by leptin deficiency upregulated the expression of TLR1-9 and TLR11-13 in murine adipose tissues. These upregulated expressions of TLRs in the expanded adipose tissues of obese animals were linked with downstream NF*κ*B, IRFs, and STAT-1 activation and upregulated cytokines and chemokines expression via MyD88-dependent and -independent cascades in the adipose tissues of the obese mice [74].

Accumulated evidence has indicated the anti-inflammatory role of adiponectin. Plasma adiponectin levels are negatively correlated with CRP levels in obese or diabetic patients, and low adiponectin levels are associated with higher CRP levels in nondiabetic or healthy subjects [75]. Adiponectin-deficient mice have higher levels of *Tnf *mRNA in adipose tissue and TNF protein in the blood, and these increases were restored to normal levels on administration of adiponectin [76]. In 2000, it was first mentioned that adiponectin could negatively regulate the functions of the macrophages [77]. Further study showed that globular adiponectin (gAcrp) could inhibit TLR-mediated NF-**κ**B signaling in mouse macrophage through binding to the AdipoR1 receptor [53]. In addition, Huang et al. mentioned a complex integrated mechanism that macrophage responded to gAcrp [78]. gAcrp initially activated signaling pathways considered to be proinflammatory, with a subsequent increase in the expression of the potent, anti-inflammatory cytokine, and IL-10. Increased IL-10 expression was ultimately required for the suppression of TLR4-mediated signaling by gAcrp.

## 5. Obesity: A Newly Established Risk for HCC

Hepatitis B carrier state, chronic hepatitis C virus infection and alcoholism have been established as the main risk factors of HCC for decades. However, it is estimated that 5% to 30% of patients with HCC still lack a readily identifiable risk factor [79]. In 2004, Caldwell et al. emphasized the importance of obesity in the occurrence of HCC [80]. Meanwhile, they also mentioned that the intimate relationship between type 2 diabetes and HCC should not be neglected. Since then, accumulated studies have attempted to delineate how obesity and its subsequent metabolic disease status contribute to the development of HCC. 

In recent years, the prevalence of obesity has progressively increased worldwide and has become a major health issue. The World Health Organization estimates that by 2015, approximately 700 million adults will be obese [81]. Obesity is a medical condition in which excess body fat has accumulated to the extent that it may cause a number of health problems and lead to reduced life expectancy. Obesity is associated with a broad range of comorbidities, also including cancer [82]. One epidemiological report points out that 15–20% of all cancer deaths in the United States can be attributed to overweight and obesity [83]. Furthermore, it is found that the relative risk of dying from liver cancer was 1.68 times higher among women with a baseline BMI ≥ 35 kg/m^2^ and was 4.52 times higher for men with a similarly increased BMI compared with the reference groups with baseline BMIs of 18.5 to 24.9 kg/m^2^. To date, nonalcoholic fatty liver disease (NAFLD) and diabetes are the most common obesity-related comorbidities which may explain the majority of cryptogenic HCC without known underlying chronic liver disease [84, 85]. 

### 5.1. NAFLD and HCC

When the mechanisms linking obesity to the development of HCC are discussed, nonalcoholic fatty liver disease (NAFLD) might be the possible direction. NAFLD encompasses a clinicopathologic spectrum of disease ranging from isolated hepatic steatosis to nonalcoholic steatohepatitis (NASH), the more aggressive form of fatty liver disease, which can progress to cirrhosis and its associated complications, including hepatic failure and HCC [86]. NASH may account for a large proportion of idiopathic or cryptogenic cirrhosis (CC), which predisposes these patients to the development of HCC [87].

The development of NASH is associated with oxidative stress and the release of reactive oxygen species (ROS). An insulin resistant obese mouse model demonstrated that ROS production is increased in the mitochondria of hepatocytes with fatty infiltration, and that oxidative stress may be implicated in hepatic hyperplasia, which is a predisposing factor for cancer development [88]. HCC development in NASH may be partially mediated by increased release of inflammatory and inhibitory cytokines such as TNF-*α*, IL-6, and NF-*κ*B. Evidence suggests a complex molecular interplay related to these inflammatory cytokines that leads to hepatocyte death, compensatory proliferation, and ultimately carcinogenesis [89]. A recent study by Luedde et al. demonstrated that inhibition of NF-*κ*B in mouse livers induced steatohepatitis and ultimately HCC by sensitizing hepatocytes to spontaneous apoptosis [90].

The c-Jun amino-terminal kinase 1 (JNK1) has also recently been linked to obesity, insulin resistance, NASH, and HCC development. JNK1 is a ubiquitously expressed, mitogen-activated protein kinase. Obesity is associated with abnormally elevated JNK activity. Free fatty acids, TNF-*α*, and ROS released in the setting of hyperinsulinemia are all potent activators of JNK [91]. More recently, definitive evidence has revealed a significant relationship between sustained JNK activation and the development of HCC [92]. In one study, 56% of HCC tissue samples demonstrated elevated JNK1 activity relative to the case-matched noncancerous liver tissue [93]. This sustained overactivation of JNK1 leads to an aberrant increase in several genes important for hepatocyte proliferation [94].

### 5.2. Diabetes Mellitus and HCC

Large population-based cohort studies from Sweden, Denmark, and Greece demonstrate a 1.86- to 4-fold increase in risk of HCC among patients with diabetes [95–97]. The risk of HCC with diabetes remained elevated even after excluding patients who were subsequently diagnosed with HCV, HBV, alcohol use, and/or fatty liver disease at any time during the followup [98]. Interestingly, the risk of HCC from diabetes may be decreased with the use of statins. A total of 1303 cases and 5212 controls were compared in a nested, matched, case-control study in patients with diabetes given the known higher risk of developing HCC. The study demonstrated a risk reduction range between 25% and 40% for the development of HCC in diabetic patients who were prescribed statins [99]. Therefore, diabetes is clearly established as an independent risk factor for HCC.

## 6. The Involvement of Adipokines in Liver Carcinogenesis

Since NAFLD, the hepatic presentation of the metabolic syndrome has been established as another risk factor to explain the majority of “cryptogenic” HCC, the association of adipokines with HCC development has been widely studied [100]. In 2006, Wang et al., who evaluated the expression of leptin and its receptor in HCC specimens by immunostaining, further correlated the expression profile with Ki-67 expression, intratumor MVD, and overall survival and provided clinical evidence on the prognostic roles of leptin and OBR in HCC patients [101, 102]. First, OBR expression was inversely correlated with vascular invasion of HCC. Furthermore, high leptin expression was associated with better survival in patients with HCC and treated postoperatively with medroxyprogesterone acetate, a synthetic variant of human progesterone. As a result, it was suggested that both high leptin and OBR expression in HCC tissues could predict better overall survival. A number of studies have defined the effects of leptin on HCC progression. Ribatti et al., using the CAM assay, demonstrated the involvement of leptin/leptin receptor in angiogenesis and tumor progression of HCC [103]. Similar result in one in vitro assay suggested that leptin-mediated neovascularization coordinated with VEGF plays an important role in the development of liver fibrosis and hepatocarcinogenesis in NASH [104]. In addition, recent study further demonstrated that leptin promotes hepatocytic cell cycle progression by upregulation of cyclin D1 and reduces programmed cell death by downregulation of Bax through a JAK2-PI3K/Akt-MEK/ERK1/2 cascaded pathway [105]. 

Adiponectin interacts with at least two known cellular receptors (AdipoR1 and AdipoR2). AdipoR1 is expressed widely in mice, whereas AdipoR2 is expressed mainly in the liver [106]. Activation of AdipoR1 and/or AdipoR2 by adiponectin stimulates the activation of peroxisome-proliferator-activated receptor-*α* (PPAR*α*), AMP-activated protein kinase (AMPK), and p38 mitogen-activated protein kinase. Adiponectin regulates the expression of several pro- and anti-inflammatory cytokines. Recent in vitro assay demonstrated that adiponectin could increase apoptosis of HCC cells through increased phosphorylation of c-Jun-N-terminal kinase (JNK) and subsequent activation of caspase-3 [107]. Moreover, analysis of adiponectin expression levels in tissue microarray of human HCC patients revealed an inverse correlation of adiponectin expression with tumor size. Another study provided some important information about the suppression of adiponectin on the oncogenic actions of leptin in HCC [108]: (1) Adiponectin treatment reduced leptin-induced Stat3 and Akt phosphorylation and increased suppressor of cytokine signaling (SOCS3), a physiologic negative regulator of leptin signal transduction. (2) Adiponectin significantly reduced leptin-induced tumor burden in nude mice.

## 7. Evidence Indicating the Potential Interplay between Adipokines and TLRs in HCC Development

As previously mentioned, TRLs have been implicated in the HCC carcinogenesis initiated by chronic inflammation of different etiologies, including HBV, HCV, and alcohol [31]. Obesity, a state of chronic inflammation, has been newly established as a potential promoter of HCC development, so the adipokines become the potential candidates to explain the link [84, 85]. In addition to the metabolic role, each adipokine also individually exerts its proinflammatory or anti-inflammatory effect in a number of obesity-related human disease, including HCC. In the context, both of TLRs and adipokines function as the crucial link between chronic inflammation and HCC; it is assumable that there is some interplay between TLRs and adipokines in the hepatocarcinogenesis. Herein, previous evidence could provide several clues to explain this potential interaction. First, TLRs have been demonstrated to be expressed in different types of hepatic tissues (such as hepatocytes, Kupffer cells, and stellate cells) [21], and accumulating data have unveiled the effects of diverse adipokines in these hepatic tissues [100, 107, 108]. Second, TLRs and adipokine receptors have several common intracellular targets (e.g., NF-*κ*B and mitogen-activated protein kinases), so it will not be surprising to find out some interaction between these two receptor families in the future. Third, a number of adipokines (e.g., TNF and IL-6), which are released by obese adipose tissue during weight gain, greatly contribute to the recruitment of macrophages, which have been demonstrated to express a range of TLRs. Lastly, recent studies have reported that a variety of TLRs are expressed in the adipocytes, and several adipokines modulate the inflammation through TLR pathways.

## 8. Conclusions and Future Directions

Adipose tissue has recently been considered as an endocrine organ, because adipocytes can produce a variety of bioactive hormones, collectively referred to as “adipokines”. Adipokines are thought to provide an important link between obesity, insulin resistance, and related inflammatory disorders. In addition to adipocytes, adipose tissues also contain endothelial cells, fibroblasts, leukocytes, and, most importantly, macrophages. Once macrophages are present and active in the adipose tissue during weight gain, they, together with adipokines, might perpetuate a vicious cycle of macrophage recruitment and production of proinflammatory cytokines. Therefore, obesity has been considered as a state of chronic low-grade inflammation. 

TLRs, one type of pattern recognition receptor, can be engaged by both exogenous ligands (derived from pathogens) and endogenous ligands (products of cellular injury) and consequently lead to the expression of diverse inflammatory genes. It has been demonstrated that TLRs are expressed on all major subsets of liver cells, including hepatocytes, Kupffer cells, hepatic stellate cells, and so on. These receptors have been implicated in chronic liver inflammation of different etiologies (HBV, HCV, toxic liver injury, etc.), all of which might predispose to the development of liver cirrhosis and HCC. Recently, obesity has been advocated as another new risk factor for HCC and could explain the majority of “cryptogenic” HCC. In fact, TLRs have been also considered as a key link between inflammation and metabolism.

Accumulating data have been demonstrating their respective contribution of adipokines and TLRs to the carcinogenesis of HCC. In context of similar potential in linking inflammation to metabolism, it is assumable that the interplay between adipokines and TLRs might exert some substantial effects on the development of HCC. Also, a better understanding in the interplay between adipokines and TLRs will shed light in a new therapeutic alternative in HCC.

## References

[1] Gay NJ, Keith FJ (1991). Drosophila Toll and IL-1 receptor. *Nature*.

[2] Medzhitov R (2001). Toll-like receptors and innate immunity. *Nature Reviews Immunology*.

[3] Akira S, Uematsu S, Takeuchi O (2006). Pathogen recognition and innate immunity. *Cell*.

[4] Janeway CA (1989). Approaching the asymptote? Evolution and revolution in immunology. *Cold Spring Harbor Symposia on Quantitative Biology*.

[5] Bell JK, Mullen GE, Leifer CA, Mazzoni A, Davies DR, Segal DM (2003). Leucine-rich repeats and pathogen recognition in Toll-like receptors. *Trends in Immunology*.

[6] O’Neill LA, Bowie AG (2007). The family of five: TIR-domain-containing adaptors in Toll-like receptor signalling. *Nature Reviews Immunology*.

[7] So EY, Ouchi T (2010). The application of Toll like receptors for cancer therapy. *International Journal of Biological Sciences*.

[8] Medzhitov R, Preston-Hurlburt P, Kopp E (1998). MyD88 is an adaptor protein in the hToll/IL-1 receptor family signaling pathways. *Molecular Cell*.

[9] Yamamoto M, Sato S, Hemmi H (2002). Essential role for TIRAP in activation of the signalling cascade shared by TLR2 and TLR4. *Nature*.

[10] Yamamoto M, Sato S, Hemmi H (2003). Role of adaptor TRIF in the MyD88-independent toll-like receptor signaling pathway. *Science*.

[11] Kagan JC, Su T, Horng T, Chow A, Akira S, Medzhitov R (2008). TRAM couples endocytosis of Toll-like receptor 4 to the induction of interferon-beta. *Nature Immunology*.

[12] Mink M, Fogelgren B, Olszewski K, Maroy P, Csiszar K (2001). A novel human gene (SARM) at chromosome 17q11 encodes a protein with a SAM motif and structural similarity to Armadillo/beta-catenin that is conserved in mouse, Drosophila, and Caenorhabditis elegans. *Genomics*.

[13] Burns K, Janssens S, Brissoni B, Olivos N, Beyaert R, Tschopp J (2003). Inhibition of interleukin 1 receptor/toll-like receptor signaling through the alternatively spliced, short form of MyD88 is due to its failure to recruit IRAK-4. *Journal of Experimental Medicine*.

[14] Chen ZJ (2005). Ubiquitin signalling in the NF-kappaB pathway. *Nature Cell Biology*.

[15] Alexopoulou L, Holt AC, Medzhitov R, Flavell RA (2001). Recognition of double-stranded RNA and activation of NF-kappaB by Toll-like receptor 3. *Nature*.

[16] Yamamoto M, Sato S, Hemmi H (2003). TRAM is specifically involved in the Toll-like receptor 4-mediated MyD88-independent signaling pathway. *Nature Immunology*.

[17] Phipps S, Lam CE, Foster PS, Matthaei KI (2007). The contribution of toll-like receptors to the pathogenesis of asthma. *Immunology and Cell Biology*.

[18] Anders HJ, Schlöndorff D (2007). Toll-like receptors: emerging concepts in kidney disease. *Current Opinion in Nephrology and Hypertension*.

[19] Drexler SK, Foxwell BM (2010). The role of Toll-like receptors in chronic inflammation. *International Journal of Biochemistry and Cell Biology*.

[20] Balkwill F, Mantovani A (2001). Inflammation and cancer: back to Virchow?. *The Lancet*.

[21] Testro AG, Visvanathan K (2009). Toll-like receptors and their role in gastrointestinal disease. *Journal of Gastroenterology and Hepatology*.

[22] Liu S, Gallo DJ, Green AM (2002). Role of toll-like receptors in changes in gene expression and NF-kappa B activation in mouse hepatocytes stimulated with lipopolysaccharide. *Infection and Immunity*.

[23] Seki E, Tsutsui H, Nakano H (2001). Lipopolysaccharide-induced IL-18 secretion from murine Kupffer cells independently of myeloid differentiation factor 88 that is critically involved in induction of production of IL-12 and IL-1beta. *Journal of Immunology*.

[24] Thobe BM, Frink M, Hildebrand F (2007). The role of MAPK in Kupffer cell Toll-like receptor (TLR) 2-, TLR4-, and TLR9-mediated signaling following trauma-hemorrhage. *Journal of Cellular Physiology*.

[25] Paik YH, Schwabe RF, Bataller R, Russo MP, Jobin C, Brenner DA (2003). Toll-like receptor 4 mediates inflammatory signaling by bacterial lipopolysaccharide in human hepatic stellate cells. *Hepatology*.

[26] Watanabe A, Hashmi A, Gomes DA (2007). Apoptotic hepatocyte DNA inhibits hepatic stellate cell chemotaxis via toll-like receptor 9. *Hepatology*.

[27] Harada K, Isse K, Sato Y, Ozaki S, Nakanuma Y (2006). Endotoxin tolerance in human intrahepatic biliary epithelial cells is induced by upregulation of IRAK-M. *Liver International*.

[28] Uhrig A, Banafsche R, Kremer M (2005). Development and functional consequences of LPS tolerance in sinusoidal endothelial cells of the liver. *Journal of Leukocyte Biology*.

[29] Isogawa M, Robek MD, Furuichi Y, Chisari FV (2005). Toll-like receptor signaling inhibits hepatitis B virus replication in vivo. *Journal of Virology*.

[30] Wu J, Lu M, Meng Z (2007). Toll-like receptor-mediated control of HBV replication by nonparenchymal liver cells in mice. *Hepatology*.

[31] Szabo G, Dolganiuc A, Mandrekar P (2006). Pattern recognition receptors: a contemporary view on liver diseases. *Hepatology*.

[32] Mandrekar P, Szabo G (2009). Signalling pathways in alcohol-induced liver inflammation. *Journal of Hepatology*.

[33] Park HS, Jung HY, Park EY, Kim J, Lee WJ, Bae YS (2004). Cutting edge: direct interaction of TLR4 with NADPH oxidase 4 isozyme is essential for lipopolysaccharide-induced production of reactive oxygen species and activation of NF-kappa B. *Journal of Immunology*.

[34] Hotamisligil GS (2006). Inflammation and metabolic disorders. *Nature*.

[35] Shoelson SE, Lee J, Goldfine AB (2006). Inflammation and insulin resistance. *Journal of Clinical Investigation*.

[36] Visser M, Bouter LM, McQuillan GM, Wener MH, Harris TB (1999). Elevated C-reactive protein levels in overweight and obese adults. *Journal of the American Medical Association*.

[37] Pradhan AD, Manson JE, Rifai N, Buring JE, Ridker PM (2001). C-reactive protein, interleukin 6, and risk of developing type 2 diabetes mellitus. *Journal of the American Medical Association*.

[38] Galic S, Oakhill JS, Steinberg GR (2010). Adipose tissue as an endocrine organ. *Molecular and Cellular Endocrinology*.

[39] Trujillo ME, Scherer PE (2006). Adipose tissue-derived factors: impact on health and disease. *Endocrine Reviews*.

[40] Zhang Y, Proenca R, Maffei M, Barone M, Leopold L, Friedman JM (1994). Positional cloning of the mouse obese gene and its human homologue. *Nature*.

[41] Friedman JM, Halaas JL (1998). Leptin and the regulation of body weight in mammals. *Nature*.

[42] Zhang F, Basinski MB, Beals JM (1997). Crystal structure of the obese protein leptin-E100. *Nature*.

[43] Santos-Alvarez J, Goberna R, Sánchez-Margalet V (1999). Human leptin stimulates proliferation and activation of human circulating monocytes. *Cellular Immunology*.

[44] Kiguchi N, Maeda T, Kobayashi Y, Fukazawa Y, Kishioka S (2009). Leptin enhances CC-chemokine ligand expression in cultured murine macrophage. *Biochemical and Biophysical Research Communications*.

[45] Zarkesh-Esfahani H, Pockley AG, Wu Z, Hellewell PG, Weetman AP, Ross RJ (2004). Leptin indirectly activates human neutrophils via induction of TNF-alpha. *Journal of Immunology*.

[46] Grunfeld C, Zhao C, Fuller J (1996). Endotoxin and cytokines induce expression of leptin, the ob gene product, in hamsters. *Journal of Clinical Investigation*.

[47] Ouchi N, Kihara S, Funahashi T, Matsuzawa Y, Walsh K (2003). Obesity, adiponectin and vascular inflammatory disease. *Current Opinion in Lipidology*.

[48] Ryo M, Nakamura T, Kihara S (2004). Adiponectin as a biomarker of the metabolic syndrome. *Circulation Journal*.

[49] Fruebis J, Tsao TS, Javorschi S (2001). Proteolytic cleavage product of 30-kDa adipocyte complement-related protein increases fatty acid oxidation in muscle and causes weight loss in mice. *Proceedings of the National Academy of Sciences of the United States of America*.

[50] Ouchi N, Kihara S, Arita Y (1999). Novel modulator for endothelial adhesion molecules: adipocyte-derived plasma protein adiponectin. *Circulation*.

[51] Hosogai N, Fukuhara A, Oshima K (2007). Adipose tissue hypoxia in obesity and its impact on adipocytokine dysregulation. *Diabetes*.

[52] Wolf AM, Wolf D, Rumpold H, Enrich B, Tilg H (2004). Adiponectin induces the anti-inflammatory cytokines IL-10 and IL-1RA in human leukocytes. *Biochemical and Biophysical Research Communications*.

[53] Yamaguchi N, Argueta JG, Masuhiro Y (2005). Adiponectin inhibits Toll-like receptor family-induced signaling. *FEBS Letters*.

[54] Steppan CM, Bailey ST, Bhat S (2001). The hormone resistin links obesity to diabetes. *Nature*.

[55] Holcomb IN, Kabakoff RC, Chan B (2000). FIZZ1, a novel cysteine-rich secreted protein associated with pulmonary inflammation, defines a new gene family. *The EMBO Journal*.

[56] Savage DB, Sewter CP, Klenk ES (2001). Resistin / Fizz3 expression in relation to obesity and peroxisome proliferator-activated receptor-gamma action in humans. *Diabetes*.

[57] Bokarewa M, Nagaev I, Dahlberg L, Smith U, Tarkowski A (2005). Resistin, an adipokine with potent proinflammatory properties. *Journal of Immunology*.

[58] Verma S, Li SH, Wang CH (2003). Resistin promotes endothelial cell activation: further evidence of adipokine-endothelial interaction. *Circulation*.

[59] Kawanami D, Maemura K, Takeda N (2004). Direct reciprocal effects of resistin and adiponectin on vascular endothelial cells: a new insight into adipocytokine-endothelial cell interactions. *Biochemical and Biophysical Research Communications*.

[60] Fukuhara A, Matsuda M, Nishizawa M (2005). Visfatin: a protein secreted by visceral fat that mimics the effects of insulin. *Science*.

[61] Ye SQ, Simon BA, Maloney JP (2005). Pre-B-cell colony-enhancing factor as a potential novel biomarker in acute lung injury. *The American Journal of Respiratory and Critical Care Medicine*.

[62] Jia SH, Li Y, Parodo J (2004). Pre-B cell colony-enhancing factor inhibits neutrophil apoptosis in experimental inflammation and clinical sepsis. *Journal of Clinical Investigation*.

[63] Hotamisligil GS, Shargill NS, Spiegelman BM (1993). Adipose expression of tumor necrosis factor-alpha: direct role in obesity-linked insulin resistance. *Science*.

[64] Hotamisligil GS, Budavari A, Murray D, Spiegelman BM (1994). Reduced tyrosine kinase activity of the insulin receptor in obesity-diabetes. Central role of tumor necrosis factor-alpha. *Journal of Clinical Investigation*.

[65] Kern PA, Saghizadeh M, Ong JM, Bosch RJ, Deem R, Simsolo RB (1995). The expression of tumor necrosis factor in human adipose tissue. Regulation by obesity, weight loss, and relationship to lipoprotein lipase. *Journal of Clinical Investigation*.

[66] Ziccardi P, Nappo F, Giugliano G (2002). Reduction of inflammatory cytokine concentrations and improvement of endothelial functions in obese women after weight loss over one year. *Circulation*.

[67] Fried SK, Bunkin DA, Greenberg AS (1998). Omental and subcutaneous adipose tissues of obese subjects release interleukin-6: depot difference and regulation by glucocorticoid. *Journal of Clinical Endocrinology and Metabolism*.

[68] Gregor MF, Hotamisligil GS (2011). Inflammatory mechanisms in obesity. *Annual Review of Immunology*.

[69] Lin Y, Lee H, Berg AH, Lisanti MP, Shapiro L, Scherer PE (2000). The lipopolysaccharide-activated Toll-like receptor (TLR)-4 induces synthesis of the closely related receptor TLR-2 in adipocytes. *Journal of Biological Chemistry*.

[70] Cho HH, Bae YC, Jung JS (2006). Role of toll-like receptors on human adipose-derived stromal cells. *Stem Cells*.

[71] Creely SJ, McTernan PG, Kusminski CM (2007). Lipopolysaccharide activates an innate immune system response in human adipose tissue in obesity and type 2 diabetes. *The American Journal of Physiology—Endocrinology and Metabolism*.

[72] La Cava A, Matarese G (2004). The weight of leptin in immunity. *Nature Reviews Immunology*.

[73] Batra A, Pietsch J, Fedke I (2007). Leptin-dependent toll-like receptor expression and responsiveness in preadipocytes and adipocytes. *The American Journal of Pathology*.

[74] Fresno M, Alvarez R, Cuesta N (2011). Toll-like receptors, inflammation, metabolism and obesity. *Archives of Physiology and Biochemistry*.

[75] Ouchi N, Kihara S, Funahashi T (2003). Reciprocal association of C-reactive protein with adiponectin in blood stream and adipose tissue. *Circulation*.

[76] Maeda N, Shimomura I, Kishida K (2002). Diet-induced insulin resistance in mice lacking adiponectin/ACRP30. *Nature Medicine*.

[77] Yokota T, Oritani K, Takahashi I (2000). Adiponectin, a new member of the family of soluble defense collagens, negatively regulates the growth of myelomonocytic progenitors and the functions of macrophages. *Blood*.

[78] Huang H, Park PH, McMullen MR, Nagy LE (2008). Mechanisms for the anti-inflammatory effects of adiponectin in macrophages. *Journal of Gastroenterology and Hepatology*.

[79] Caldwell SH, Oelsner DH, Iezzoni JC, Hespenheide EE, Battle EH, Driscoll CJ (1999). Cryptogenic cirrhosis: clinical characterization and risk factors for underlying disease. *Hepatology*.

[80] Caldwell SH, Crespo DM, Kang HS, Al-Osaimi AM (2004). Obesity and hepatocellular carcinoma. *Gastroenterology*.

[81] WHO Obesity and overweight. http://www.who.int/mediacentre/factsheets/fs311/en/index.html.

[82] Dixon JB (2010). The effect of obesity on health outcomes. *Molecular and Cellular Endocrinology*.

[83] Calle EE, Rodriguez C, Walker-Thurmond K, Thun MJ (2003). Overweight, obesity, and mortality from cancer in a prospectively studied cohort of U.S. Adults. *The New England Journal of Medicine*.

[84] Starley BQ, Calcagno CJ, Harrison SA (2010). Nonalcoholic fatty liver disease and hepatocellular carcinoma: a weighty connection. *Hepatology*.

[85] Polesel J, Zucchetto A, Montella M (2009). The impact of obesity and diabetes mellitus on the risk of hepatocellular carcinoma. *Annals of Oncology*.

[86] Ong JP, Younossi ZM (2007). Epidemiology and natural history of NAFLD and NASH. *Clinics in Liver Disease*.

[87] Bugianesi E, Leone N, Vanni E (2002). Expanding the natural history of nonalcoholic steatohepatitis: from cryptogenic cirrhosis to hepatocellular carcinoma. *Gastroenterology*.

[88] Ishii H, Horie Y, Ohshima S (2009). Eicosapentaenoic acid ameliorates steatohepatitis and hepatocellular carcinoma in hepatocyte-specific Pten-deficient mice. *Journal of Hepatology*.

[89] Sakurai T, Maeda S, Chang L, Karin M (2006). Loss of hepatic NF-kappa B activity enhances chemical hepatocarcinogenesis through sustained c-Jun N-terminal kinase 1 activation. *Proceedings of the National Academy of Sciences of the United States of America*.

[90] Luedde T, Beraza N, Kotsikoris V (2007). Deletion of NEMO/IKKgamma in liver parenchymal cells causes steatohepatitis and hepatocellular carcinoma. *Cancer Cell*.

[91] Hirosumi J, Tuncman G, Chang L (2002). A central role for JNK in obesity and insulin resistance. *Nature*.

[92] Chang Q, Zhang Y, Beezhold KJ (2009). Sustained JNK1 activation is associated with altered histone H3 methylations in human liver cancer. *Journal of Hepatology*.

[93] Hui L, Zatloukal K, Scheuch H, Stepniak E, Wagner EF (2008). Proliferation of human HCC cells and chemically induced mouse liver cancers requires JNK1-dependent p21 downregulation. *Journal of Clinical Investigation*.

[94] Chen F, Beezhold K, Castranova V (2009). JNK1, a potential therapeutic target for hepatocellular carcinoma. *Biochimica et Biophysica Acta*.

[95] Adami HO, Chow WH, Nyrén O (1996). Excess risk of primary liver cancer in patients with diabetes mellitus. *Journal of the National Cancer Institute*.

[96] Wideroff L, Gridley G, Mellemkjaer L (1997). Cancer incidence in a population based cohort of patients hospitalized with diabetes mellitus in Denmark. *Journal of the National Cancer Institute*.

[97] Lagiou P, Kuper H, Stuver SO, Tzonou A, Trichopoulos D, Adami HO (2000). Role of diabetes mellitus in the etiology of hepatocellular carcinoma. *Journal of the National Cancer Institute*.

[98] El-Serag HB, Tran T, Everhart JE (2004). Diabetes increases the risk of chronic liver disease and hepatocellular carcinoma. *Gastroenterology*.

[99] El-Serag HB, Johnson ML, Hachem C, Morgana RO (2009). Statins are associated with a reduced risk of hepatocellular carcinoma in a large cohort of patients with diabetes. *Gastroenterology*.

[100] Wang SN, Lee KT, Ker CG (2010). Leptin in hepatocellular carcinoma. *World Journal of Gastroenterology*.

[101] Wang SN, Yeh YT, Yang SF, Chai CY, Lee KT (2006). Potential role of leptin expression in hepatocellular carcinoma. *Journal of Clinical Pathology*.

[102] Wang SN, Chuang SC, Yeh YT (2006). Potential prognostic value of leptin receptor in hepatocellular carcinoma. *Journal of Clinical Pathology*.

[103] Ribatti D, Belloni AS, Nico B, Di Comite M, Crivellato E, Vacca A (2008). Leptin-leptin receptor are involved in angiogenesis in human hepatocellular carcinoma. *Peptides*.

[104] Kitade M, Yoshiji H, Kojima H (2006). Leptin-mediated neovascularization is a prerequisite for progression of nonalcoholic steatohepatitis in rats. *Hepatology*.

[105] Chen C, Chang YC, Liu CL, Liu TP, Chang KJ, Guo IC (2007). Leptin induces proliferation and anti-apoptosis in human hepatocarcinoma cells by up-regulating cyclin D1 and down-regulating Bax via a Janus kinase 2-linked pathway. *Endocrine-Related Cancer*.

[106] Yamauchi T, Kamon J, Ito Y (2003). Cloning of adiponectin receptors that mediate antidiabetic metabolic effects. *Nature*.

[107] Saxena NK, Fu PP, Nagalingam A (2010). Adiponectin modulates C-Jun N-terminal kinase and mammalian target of rapamycin and inhibits hepatocellular carcinoma. *Gastroenterology*.

[108] Sharma D, Wang J, Fu PP (2007). Adiponectin antagonizes the oncogenic actions of leptin in hepatocellular carcinogenesis. *Endocrine-Related Cancer*.

